# Molecular Dynamics Simulations of Transmembrane Cyclic Peptide Nanotubes Using Classical Force Fields, Hydrogen Mass Repartitioning, and Hydrogen Isotope Exchange Methods: A Critical Comparison

**DOI:** 10.3390/ijms23063158

**Published:** 2022-03-15

**Authors:** Daniel Conde, Pablo F. Garrido, Martín Calvelo, Ángel Piñeiro, Rebeca Garcia-Fandino

**Affiliations:** 1 Center for Research in Biological Chemistry and Molecular Materials, Departamento de Química Orgánica, Universidade de Santiago de Compostela, Campus Vida s/n, 15782 Santiago de Compostela, Spain; daniel.conde@rai.usc.es (D.C.); martin.calvelo.souto@usc.es (M.C.); 2 Departamento de Física Aplicada, Facultade de Física, Universidade de Santiago de Compostela, 15782 Santiago de Compostela, Spain; pablo.fernandez@usc.es; 3 Departament de Química Inorgánica i Orgànica and Institut de Química Teòrica i Computacional (IQTCUB), Universitat de Barcelona, 08028 Barcelona, Spain; 4 CIQUP, Centro de Investigação em Química, Departamento de Química e Bioquímica, Faculdade de Ciências, Universidade do Porto, 4196-007 Porto, Portugal

**Keywords:** cyclic peptides, antimicrobial peptides, self-assembled peptide nanotubes, molecular dynamics, force field, hydrogen mass repartitioning, hydrogen isotope exchange

## Abstract

Self-assembled cyclic peptide nanotubes with alternating *D*- and *L*-amino acid residues in the sequence of each subunit have attracted a great deal of attention due to their potential for new nanotechnology and biomedical applications, mainly in the field of antimicrobial peptides. Molecular dynamics simulations can be used to characterize these systems with atomic resolution at different time scales, providing information that is difficult to obtain via wet lab experiments. However, the performance of classical force fields typically employed in the simulation of biomolecules has not yet been extensively tested with this kind of highly constrained peptide. Four different classical force fields (AMBER, CHARMM, OPLS, and GROMOS), using a nanotube formed by eight *D*,*L*-α-cyclic peptides inserted into a lipid bilayer as a model system, were employed here to fill this gap. Significant differences in the pseudo-cylindrical cavities formed by the nanotubes were observed, the most important being the diameter of the nanopores, the number and location of confined water molecules, and the density distribution of the solvent molecules. Furthermore, several modifications were performed on GROMOS54a7, aiming to explore acceleration strategies of the MD simulations. The hydrogen mass repartitioning (HMR) and hydrogen isotope exchange (HIE) methods were tested to slow down the fastest degrees of freedom. These approaches allowed a significant increase in the time step employed in the equation of the motion integration algorithm, from 2 fs up to 5–7 fs, with no serious changes in the structural and dynamical properties of the nanopores. Subtle differences with respect to the simulations with the unmodified force fields were observed in the concerted movements of the cyclic peptides, as well as in the lifetime of several H-bonds. All together, these results are expected to contribute to better understanding of the behavior of self-assembled cyclic peptide nanotubes, as well as to support the methods tested to speed up general MD simulations; additionally, they do provide a number of quantitative descriptors that are expected to be used as a reference to design new experiments intended to validate and complement computational studies of antimicrobial cyclic peptides.

## 1. Introduction

**Figure 1:**
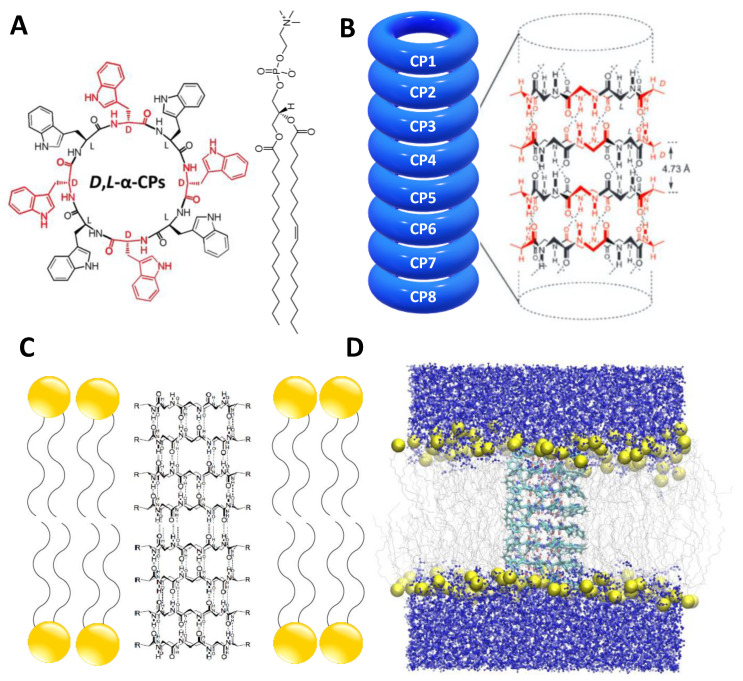
(**A**). Structures of the *D*,*L*-α-CP employed in this study, *c*-[(*L*-Trp-*D*-Trp-)4], and POPC. *L*-residues are represented in black and *D*-residues in red. (**B**). Antiparallel disposition of the CPs in the *D*,*L*-α-SCPN; note that the *D* (red) and *L* (black) residues are facing one another. (**C**). Model of the SCPN inserted into a lipid bilayer. (**D**). Initial structure used for all of the MD simulations, where the SCPN is inserted into a lipid bilayer; water molecules are on both sides of the membrane, while the nanotube cavity and the hydrophobic core of the bilayer are dry; yellow spheres correspond to the POPC phosphorous atoms.

Self-assembled cyclic peptide nanotubes (SCPNs) are considered to be one of the most intriguing building blocks employed in nanotechnological applications; they have attracted special attention due to the ability to modify their structure and, thus, their functional properties. This makes them well suited to being used in numerous applications, such as electronic devices, artificial photosystems, photoresponsive materials, biosensors, antimicrobials and antiviral agents, selective transmembrane transport channels, catalysis, and drug delivery [1,2]. The first SCPNs were devised in 1974 [3], but they could not be synthesized until 1993 [4]. These SCPNs were composed of *D*- and *L*-α-amino acids adopting a flat conformation, with the amide groups (NH and CO) lying perpendicular to the plane of the cyclic peptides (CPs). This enabled the formation of hydrogen bonds (H-bonds) between adjacent CP units (Figure 1) [3,4,5]. Such a special arrangement leaves an empty cavity in the interior of the assembly, with all of the side chains exposed on the external surface of the cylindrical structure. One of the main advantages of these nanostructures is the simplicity with which their internal and external properties can be modified based only on the selection of the appropriate CP sequence: the inner diameter of the SCPNs is determined by the number of residues, whereas their outer surface properties depend on the amino acid side chains. In this sense, SCPNs with appropriate hydrophobic sequences have emerged as attractive transmembrane channel mimics designed to replicate the specific functions of natural transport systems, in terms of affinity, efficiency, stability, and selectivity [1,2,6,7,8,9,10].

Structural and dynamic information at atomic resolution is hard to obtain for these systems. Most of the available experiments provide values for macroscopic observables that are not easy to connect with the structure of the SCPNs [5,6,11,12]. Molecular dynamics (MD) simulations can potentially play a key role in the description and characterization of SCPNs [13]; they offer a unique opportunity to reveal their mechanisms of action at spatial and temporal scales that are difficult to observe experimentally, thus working as a computational nanoscale microscope for these systems. The reliability of MD simulations largely depends on two critical factors: the accuracy of the force field used to describe interactions within the simulated system, and the lack of sampling arising from the finite length of the simulations. Over the past several decades, a number of force fields—including AMBER, CHARMM, OPLS, GROMOS, and even novel machine learning force fields—have been developed and widely used for simulations of bimolecular systems [14,15,16]. The performance of most of these force fields has been extensively tested on linear peptide and protein systems [17,18,19,20], but it remains to be evaluated on highly constrained systems such as cyclic peptides. Some approaches have been made in this direction in order to reproduce the conformational properties of a model CP [21,22,23], but there is a significant literature gap in the simulation of SCPNs with different force fields. Peptide nanotubes composed of *D*,*L*-α-CPs have been simulated using mainly the CHARMM [24,25,26,27,28] and AMBER [29,30,31] force fields. However, an exhaustive force field comparison is still missing, and is crucial in order to evaluate their influence on the structural and dynamic properties of these systems.

In addition to the need to validate the results obtained from different force fields, the length of the trajectories is another critical limitation in MD simulations. For MD simulations of large biomolecules in explicit solvents with atomic resolution, the typical time scales of the trajectories are hundreds of nanoseconds (ns). The relatively recent development of code for GPU architecture makes the microsecond (µs) time scale accessible with reasonably modest resources [32,33,34,35]. Moreover, multiple long trajectories are always convenient for an appropriate statistical analysis. It is also worth noting that many important biomolecular processes take place on much longer time scales than those typically accessed by MD simulations. Coarse-grained (CG) models have successfully lowered the resolution of proteins from all-atom (AA) representation, opening new possibilities [36]. However, although CG force fields have been applied to the study of SCPNs composed of *D*,*L*-α-CPs [37,38], they present critical limitations in the description of these systems. The intrinsic simplification of the CG beads is not able to correctly reproduce the disposition of the amide groups (NH and CO) lying perpendicularly to the plane of the ring of these cyclic peptides. This makes it impossible to appropriately differentiate both faces of the ring (with the *D*- or *L*-α-residues pointing to different faces of the plane); thus, the parallel or antiparallel orientation of the β-sheet between the CPs cannot be considered. Consequently, the formation of H-bonds between the different subunits composing the nanotube is overestimated.

A variety of strategies can be adopted to improve the sampling in atomic resolution simulations [39,40]. The computational cost of an MD simulation and, thus, the actual time required to obtain a trajectory of a given length with a limited amount of computational resources, depends on several factors, in addition to the size and resolution of the system. The algorithms used to determine the long-range electrostatic interactions and the integration of the equation of motion are two key factors. In recent years, the particle mesh Ewald (PME) algorithm has been widely adopted to solve the first problem in an efficient way [41]. The second problem depends critically on the time step used to extrapolate the positions of the particles; this parameter should be large enough to allow the significant advance of the system along a trajectory, but short enough to prevent the collapse of the simulation. The presence of fast degrees of freedom in the atomic movements—mainly those of the lightest atoms—restricts the upper limit of the time step to keep the simulations stable. The shortest period of the movement (~10 fs) corresponds to bond stretching involving hydrogen atoms; this restrains the time step to a maximum of ~1 fs in order to have several integrations within a period, thus allowing the stability of the simulation. The 1 fs limit is commonly addressed with the introduction of restraints on the high-frequency bonds, typically using the SHAKE [42] or LINCS [43] algorithms. In this way, the time step can be increased up to 2 fs. From the mathematical algorithms, it is possible to slow down the fastest movements by artificially increasing the mass of the hydrogen atoms. This kind of manipulation requires formal justification. The hydrogen mass repartitioning (HMR) method [44], proposed in 1999, which consists of transferring ~2–3 Da of mass to each hydrogen atom from the heavy atom to which it is bonded, has already been tested for several systems, providing good results [45]. Using this method, the MD simulations were stable using time steps up to 6–7 fs, thus significantly lowering the computational cost of the trajectories. The HMR method exhibits some problems with AA force fields, in part due to the presence of methyl groups, where three hydrogen atoms are simultaneously bound to the same C atom [39]; thus, the mass transference of more than 2 Da would result in a pseudo-C atom lighter than each of the bound H atoms. This is not a problem for united atom (UA) force fields such as GROMOS, where only the polar hydrogen atoms are present. Additionally, there is a conceptual issue with the HMR method: it cannot be experimentally reproduced, or even conceived. An alternative to HMR, also intended to slow down the motion of hydrogen atoms, is to directly increase their mass, instead of transferring it from the bound heavy atom. This method has a direct experimental counterpart, since doubling the mass of H atoms is equivalent to a deuteration process, so it will be referred to as the hydrogen isotope exchange (HIE) method here. The mass change of H atoms does not represent the same problem as the HMR method for methyl groups in AA force fields. In turn, this leads to a significant mass gain of the whole molecule, which slows down its internal degrees of freedom as well as its absolute diffusion, eventually cancelling the sampling improvement triggered by the expansion of the time step. Again, the H mass increase is better suited for UA force fields, since the number of polar hydrogens in these force fields is much lower than that of total hydrogens in most biomolecules, meaning that the total mass gain of the whole molecule when increasing the mass of the polar hydrogens is relatively small. Additionally, it is known that polar hydrogens dissolved in deuterated water are more prone to be replaced by deuterium than non-polar hydrogens [46]. Thus, the mass increase of just polar hydrogens is even more realistic than that of all hydrogen atoms. We have recently tested the effect of a direct mass increase in the explicit/polar hydrogens of cyclodextrin systems under different conditions, using the UA GROMOS force field [47]. Notably, no significant structural differences in the solute or in the surrounding solvent were observed using different time steps and mass values for the hydrogen atoms [47]. Extrapolating what happens during experimental deuteration, two heavier H isotopes have also been assayed to increase the stability of the simulations at high time steps: (1) hydrogen 4, or quadium [48]—an unstable isotope of hydrogen made up of a proton and three neutrons in its nucleus, with a mass of 4.02643 Da; and (2) 7H: an isotope of the hydrogen atom made up of a nucleus of one proton and six neutrons, with a mass of 7.05275 Da [49]. The actual half-life of these two isotopes is negligible compared to the time scale of classical molecular degrees of freedom, but at least the homologous experiment to this simulation strategy can be conceived, and the simulation with deuterium replacement on the polar hydrogens is fully realistic.

In the present work, MD simulations of a preassembled nanotube formed by eight *D*,*L*-α-CPs and inserted in a lipid bilayer were performed using four classical force fields: AMBER, CHARMM, OPLS, and GROMOS (see Materials and Methods). Additionally, several HMR and HIE setups were applied to the simulated system, combined with different time step values, in order to test the impact of these strategies on the structural and dynamic properties of the target system. The main goals were to compare the performance of various classical force fields on the simulation of SCPNs, to provide quantitative descriptors intended to guide the design of new experiments, and to validate the use of non-standard methods able to speed up the MD simulations of these systems.

## 2. Materials and Methods

### 2.1. Simulation Systems and Parameters

The SCPN studied in this work is composed of eight *D*,*L*-α-CP rings, long enough to traverse a previously equilibrated 1-palmitoyl-2-oleoyl-sn-glycero-3-phosphocholine (POPC) membrane model. Each ring sequence consists of eight Trp residues, with alternating *D*- and *L*- configurations: *c*-[(*L*-Trp-*D*-Trp-)_4_] (Figure 1A). The starting geometries of the employed CPs were taken from previous works, which suggested a preferred antiparallel disposition of the β-sheet (Figure 1B) [50]. After the insertion of the nanotube into the POPC bilayer, the complete system was solvated. Water in the hydrophobic region of the membrane and inside the SCPN was removed, so that the channel was completely dry during the first step of the simulation (Figure 1D). The numbers of lipid and water molecules in the final system were 154 and 7367, respectively.

The CP was modeled using four force fields: AMBER99SB-ILDN [51], CHARMM36m [52], OPLS [53,54], and GROMOS54a7 [55]. The topologies of the homologous open peptides were created using GROMACS, and then they were modified to close the rings using a Python script specifically developed for this aim. Several versions of the GROMOS54a7 CP topology were created: (1) the reference topology using the standard mass for the polar hydrogen atoms (GROMOS); (2) another where all of the hydrogens are replaced by deuterium (H2D), i.e., just changing the mass of the hydrogen to 2 Da, while keeping the atom type with the corresponding van der Waals parameters and charge; (3) a copy of the previous one, but doubling the mass of the hydrogens again to mimic quadium (H2Q); and (4) another copy by using 7 Da for the mass of the hydrogen atoms (H2H7). Using GROMACS tools, an additional topology was generated by transferring 3 Da to every polar hydrogen from its bounded heavy atom, following the HMR protocol [44], automatically implemented in GROMACS (5) to both the CPs and the water (HMR_w_) or (6) to just the CPs (HMR). The water model used for each force field was the same used for its original parametrization. The TIP3P [56] water model was used for AMBER and CHARMM; the TIP4P [57] water model was used for OPLS simulations, while the SPC water model [58] was used for all of the simulations based on the GROMOS54a7 force field (including those using HMR, HMR_w_, H2D, H2Q, and H2H7). The parameters for POPC were taken from Lipidbook [59] and Zenodo: Slipids [60], CHARMM36 [61,62], OPLS [63,64], and GROMOS53a6 [65]. These parameters are perfectly compatible with the AMBER, CHARMM, OPLS, and GROMOS force fields, respectively, used for the CPs. All simulations were performed with the GROMACS 2019.3 [66] package. The systems were first minimized using a steepest descent algorithm. Then, an unrestrained production run of 300 ns, with a time step of 2 fs (for all force fields using the unmodified topologies), as well as with time steps of 5 fs (H2D), 6 fs (H2Q, H2H7, and HMR), and 7 fs (HMR_w_) for the modified topologies (HMR and HIE). Thus, a total of 9 trajectories were obtained: 4 using the unmodified topologies; 3 more using deuterium, quadium, and H7 instead of hydrogen, with time steps of 5, 6, and 6 fs, respectively; and 2 additional simulations using HMR just on the SCPN (HMR), and on both the SCPN and the solvent (HMR_w_), with time steps of 6 and 7 fs, respectively. The atomic coordinates were stored every 2 ps for all of the trajectories (300 ns long), regardless of the time step. A summary of the time steps employed in each case, as well as the corresponding masses of the polar atoms, is shown in Table 1.

An NPT ensemble was employed at 1 bar and at 300 K using a semi-isotropic Parrinello–Rahman barostat [67] and a V-rescale thermostat [68]. The LINCS algorithm was employed to remove bond vibrations [69]. Electrostatic interactions were calculated using the PME method, with a cutoff of 1.0 nm and a grid spacing of 0.12 nm [41]. Van der Waals interactions were calculated using a 1.0 nm radius cutoff. These simulation parameters were employed for all of the force fields, even when they did not correspond exactly to those employed for their original parameterization. The reason for this was that no CPs were employed in the development of the force field, so the changes made to the cutoff or to the specific parameters for the control of pressure or temperature are expected to have a lower impact on the results than the introduction of these new molecules. From the practical point of view, a common set of simulation parameters is convenient to facilitate the comparison between force fields. It is also worth mentioning that longer cutoffs do not necessarily imply more realistic MD simulations, as has been shown by increasing the short range cutoff in CHARMM simulations, which takes DPPC bilayers to a gel phase under conditions where they should be in a fluid phase [70].

### 2.2. Analysis of the Trajectories

VMD [71] was employed to generate snapshots and animations from trajectories. GROMACS commands were employed for some standard analyses: root-mean-square deviation (RMSD), root-mean-square fluctuation (RMSF), and H-bonds. The inner radius of the SCPN cavity was obtained using CHAP [72]. Other specific analyses were performed using specifically developed Python scripts, based mainly on the MDAnalysis [73,74], NumPy [75], Pandas [76], and Matplotlib [77] libraries. The first 80 ns of each trajectory was discarded for the calculation of all of the average properties, so as to ensure a proper equilibration for the analysis.

The normal vector to each CP (***n***) was determined by averaging the normalized cross-product of alternating vector differences between the position of each alpha carbon (Cα) and the geometrical center of the eight Cα (${r^{\prime }}_{i}=r_{i}-r_{c}$):

(1) $$n=\sum \left({r^{\prime }}_{i}\times {r^{\prime }}_{i+2}\right)/\left|{r^{\prime }}_{i}\times {r^{\prime }}_{i+2}\right|$$

Since our system is a cyclic peptide with 8 amino acids, the selection of the initial residue for this calculation was arbitrary, and the indices 7 and 8 were taken together with 1 and 2, in order to use all of the Cα atoms. This normal vector (***n***) and the center of the CP were used to determine the equation of the CP plane. The position of each Cα was then projected onto this plane, and the distance from said projection to the center was monitored along the MD simulation for each CP. Additionally, the angle between the projection of each ${r^{\prime }}_{i}$ vector on the CP plane (${r^{\prime \prime }}_{i}$) and the sum of all of the equivalent vectors for the remaining 7 Cα ($\sum {r^{\prime \prime }}_{j}$ with *j* ≠ *i*) was determined. The average values of these two magnitudes, together with their corresponding standard deviation, were taken as a measurement of the CP deformation in the radial direction, and of the angular distortion, respectively. This information was then represented in circular bar plots (see below).

Considering the previous definitions, the angle between each ${r^{\prime }}_{i}$ vector and the CP plane (δ) was also calculated, and the average values were plotted for comparison between CPs and force fields. Additionally, the distances between the centers of mass of consecutive CPs in the SCPN were represented as a function of time, in order to assess an eventual stretching or contraction of the whole structure in its longitudinal dimension.

The normal vector given by Equation (1) was also employed to determine the angle of each CP with respect to the membrane plane as a function of time. A principal component analysis of these angles was also performed to assess whether or not the tilting of the different subunits was a coordinated movement. Additionally, the average of the normal vectors for all the CPs was employed to determine the tilt angle of the whole SCPN as a function of time.

Finally, the lateral displacement of the whole SCPN at the membrane was also determined using different time windows, and the corresponding distributions were fitted to the two-dimensional random walk equation to obtain the corresponding diffusion coefficients. A detailed description of this analysis can be found in a previous work [78].

## 3. Results and Discussion

MD simulations of an SCPN composed of *c*-[(*L*-Trp-*D*-Trp-)_4_] inserted into a POPC lipid bilayer were carried out, using four different classical force fields commonly employed to simulate biomolecular systems: AMBER, CHARMM, OPLS, and GROMOS (see Materials and Methods). In parallel, several modifications of GROMOS54a7 topologies—aiming to slow down the fastest degrees of freedom and, thus, allow us to increase the time step employed for the integration of the equation of motion—were performed (see Materials and Methods). The HMR method proposed by Feenstra [44] was applied to the SCPN alone (simulation labelled as HMR), and to the SCPN and the solvent simultaneously (HMR_w_). These two simulations were stable, with time steps of 6 fs and 7 fs, respectively. In addition, the HIE method—mimicking the replacement of polar hydrogens (the only present in the UA GROMOS force field) by deuterium (H2D), quadium (H2Q), and hydrogen 7 (H2H7)—was also tested. The trajectory of H2D was stable, with a time step of 5 fs, while H2Q and H2H7 both had a time step of 6 fs. The structural stability and dynamics of the SCPN, as well as the behavior of the water molecules in the nanopore cavities, were analyzed in detail, comparing the results between the different force fields, parameterizations, and simulation conditions (treatment for the hydrogen mass and time step).

### 3.1. Force Field Dependence of the Structural Stability of the D,L-α-SCPN

**Figure 2:**
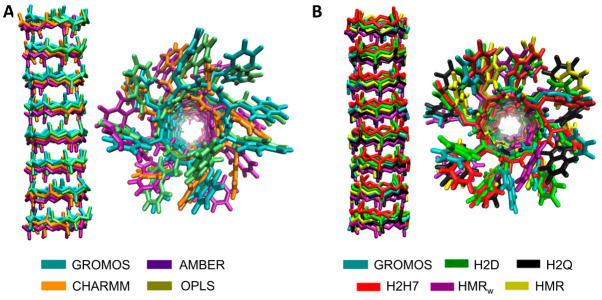
Lateral- and top-view structure alignment of the last snapshot (t = 300 ns) of the SCPN simulated with different classical force fields (**A**) and the GROMOS-modified force fields (**B**). For simplicity, only the backbone is shown for the lateral representation. Each color represents a different force field or force field parameterization. The classical GROMOS force field is included in both (**A**,**B**) as a reference.

The tubular structure of the SCPN was maintained along the whole trajectories (300 ns) with all of the force fields employed (Figure 2). RMSD and RMSF analysis, using the initial structure (after equilibration) as a reference, confirmed the stability of the channel at the quantitative level in all cases (Figures S1 and S2), with larger fluctuations corresponding to the CPs placed at the edges of the nanotubes (Figure S2). These analyses are straightforward, since they are already implemented in several MD packages and other external libraries, and are easy to understand and informative; however, they require a reference structure to which the whole nanotube should be aligned in order to obtain a quantitative number. However, the simplicity of the corresponding calculation and representation masks important information that could be revealed with a more specific analysis.

**Figure 3:**
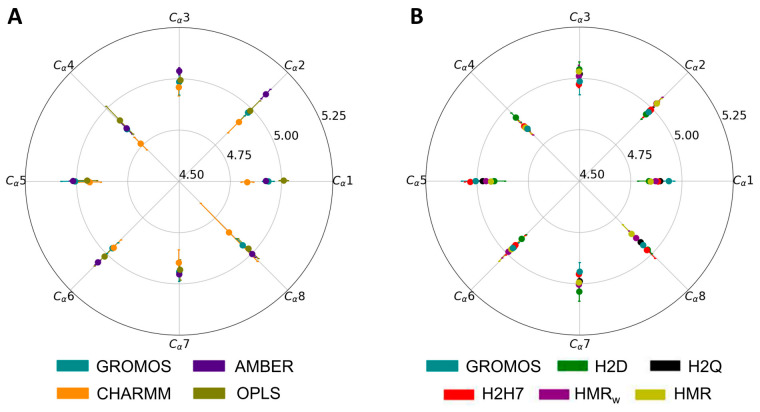
Average radial and angular contributions to the deformation of CP4 determined as explained in the Materials and Methods section, over the last 220 ns of each trajectory. The average radial distance and angle with respect to the geometrical center of the CP (in Å) are indicated in the corresponding circles. Each radial line in the circular bar plot represents the angle expected from an octagonal disposition of each Cα in the CP, while the position of the different solid circles represents the angular deviation with respect to it. The results for each force field are indicated by a different color (see legends). The results for unmodified GROMOS are reproduced in both circular bar plots (**A**,**B**) as a reference. Results for the rest of the CPs are shown in Figure S3.

The radial and angular components of the deformation and fluctuation of each CP for all of the trajectories were determined as described in the Materials and Methods section (Figure 3 and Figure S3). It can be observed that the distortion of the CPs at the edges of the SCPN is larger than that of the most internal CPs (Figure S3). The results of the different force fields and parameterizations do not exhibit important differences. Slightly larger distances between the Cα and the center of the CP were observed for the simulations with AMBER, anticipating a larger radius for the channel in this simulation (see below), while shorter distances were found for the simulations with CHARMM. The results provided by the trajectories performed using the HMR (HMR and HMR_w_ trajectories) and HIE (H2D, H2Q, and H2H7 trajectories) methods are not significantly different to those observed for other force fields.

**Figure 4:**
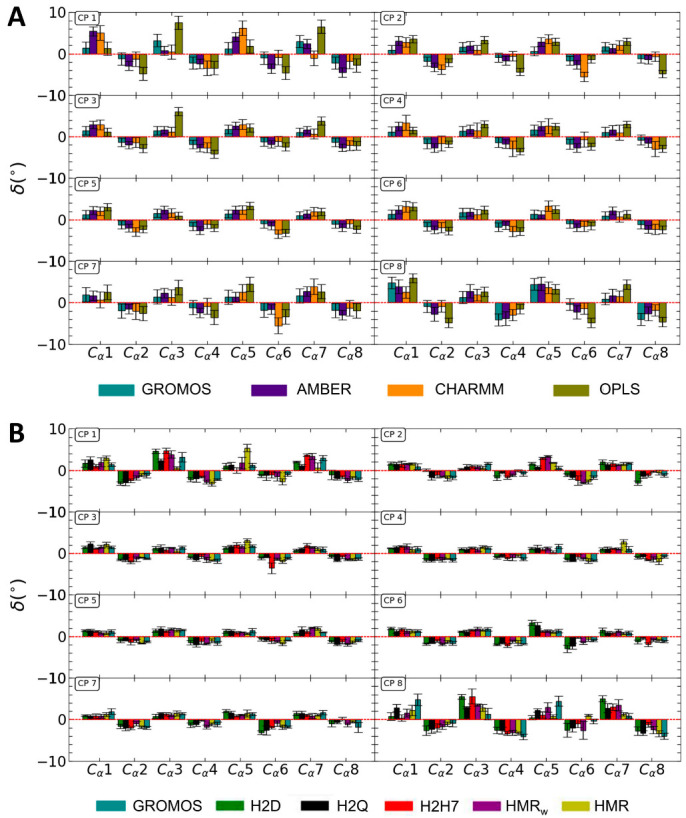
Average values of the angles between the vectors joining each Cα with the center of the CP and the CP plane for the unmodified force fields (**A**), and for the modified parameterizations of GROMOS (**B**). The uncertainties in the plot are the standard deviations obtained using the block average method [46], multiplied by 1.96 to get the values within a confidence interval of 95%.

As expected from the zigzagging structure of the CP (Figure 1B), the angles between the vectors joining each Cα with the center of the CP and the CP plane alternate between positive and negative values (Figure 4). These angles are larger, in absolute terms, for OPLS, CHARMM, and AMBER than for all of the GROMOS parameterizations. The dispersion in the angles is significantly larger for the terminal CPs of the nanotube—which are more affected by the interaction with the lipid heads and the bulk solvent—than for the internal CPs. Considering just the six central CPs, the observed average angles are (2.8 ± 0.4) for OPLS, (2.1 ± 0.2)° for CHARMM, (2.1 ± 0.1)° for AMBER and (1.4 ± 0.2)° (for all of the GROMOS parameterizations). Thus, no significant differences were observed between the simulations using the modified GROMOS topologies, while the Cα were further from the plane of the CP ring for OPLS and, to a lesser extent, for AMBER and CHARMM.

**Figure 5:**
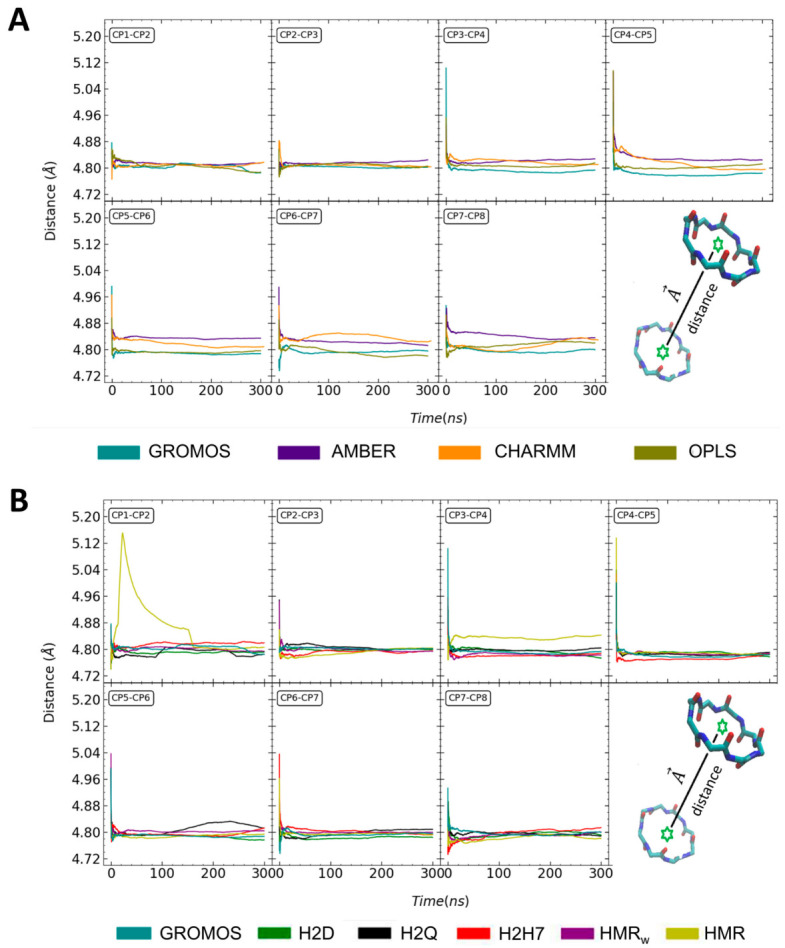
Evolution of the c.o.m. distance (in Å) between pairs of CPs along the trajectory, for the unmodified force fields (**A**), and for the modified parameterizations of GROMOS (**B**). Each color corresponds to a different force field, according to the legends.

Unlike other transmembrane channels, such as those formed by carbon nanotubes, the CPs composing the SCPN are non-covalently bonded to one another, thus allowing a greater longitudinal flexibility. The distances between the centers of mass (c.o.m.) of the CPs exhibit a slight reduction during the first few ns of all of the trajectories, and then become stable for the rest of the simulation time, with just some small and slow fluctuations of just a few hundreds of Å (Figure 5). In general, the evolution of these distances is very similar for all of the force fields and GROMOS modifications, although the subunits are slightly closer to one another for the simulations using OPLS and GROMOS than for those with AMBER and CHARMM (Figure 5). The most singular case of that is HMR, where the first two CPs experience a significant perturbation at the beginning of the trajectory, which is reflected in a strong increase in the distance between CP1 and CP2. This perturbation represents more than 100 ns, and then the distance between these two nanotube subunits converges to the same value observed for the other GROMACS parameterizations. The same trajectory reveals a significantly larger distance between CP3 and CP4 than those corresponding to the rest of the simulations. In contrast to the distance between CP1 and CP2 of the same subunit, the separation between CP3 and CP4 was stable throughout the whole trajectory. Overall, the distance between the c.o.m. of the CPs is not a highly sensitive parameter. The characteristic value for all of the force fields and CP pairs is ~4.8 Å, which for this nanotube is comparable to the average radius of the CPs.

**Figure 6:**
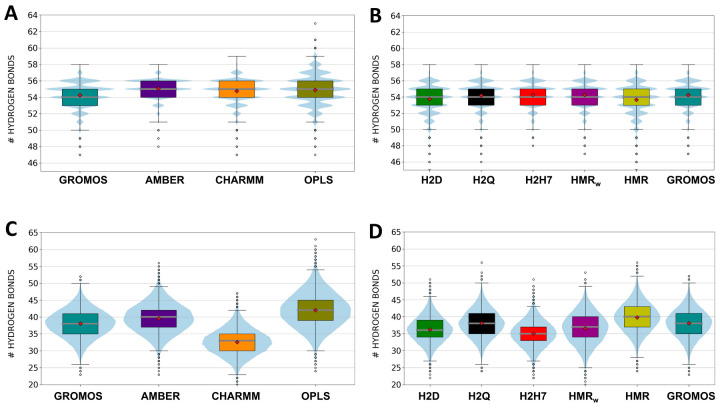
Numbers of H-bonds between CP subunits (**A**,**B**) and between the CPs and water (**C**,**D**), for the unmodified force fields (**A**,**C**), and for the modified parameterizations of GROMOS (**B**,**D**), based on the HMR and HIE methods. The grey line represents the median and the red diamond represents the mean of the distributions. The size of the boxes includes data within the second and third quartiles, so that 50% of the data are within the corresponding box. The whiskers, calculated following Tukey’s criteria [46], include data on the first and fourth quartiles. Together with the boxplot representation, the distribution of the number of H-bonds is represented by the shadows.

H-bonds between CPs play a key role in maintaining the cohesion of the SCPN structure. Differences between force fields and GROMOS parameterizations are very small (Figure 6A,B), with all of the trajectories giving an average number of H-bonds of approximately 54–55 out of a maximum of 56 possible interactions. The fraction of H-bonds larger than the maximum corresponds to those established between the Trp side chains of contiguous CPs. The values seem to be slightly lower for the simulations involving all of the GROMOS parameterizations. In order to be more specific, the H-bonds between the atoms of the backbone were also considered (Figure S4). In this case, a maximum of eight H-bonds between CP subunits can be formed. The analysis of the trajectories shows that the average number of H-bonds is very close to this maximum value for all of the simulations and between all CP pairs. The interactions between subunits involving the terminal CPs, which are in direct contact with the lipid headgroups and with the bulk solvent, seem to be slightly weaker in some trajectories. Thus, the number of H-bonds between CP7 and CP8 was ~7.5 for all of the simulations except that with OPLS, and the same happened for the interactions between CP1 and CP2, except for those using AMBER and OPLS (Figure S4).

Lipid and water molecules may also form H-bonds with CPs, eventually compromising the stability of SCPNs. The difference between force fields in the analysis of these interactions is larger than the differences observed in the numbers of H-bonds between CPs (Figure 6C,D). While the trajectory with CHARMM leads to the lowest number of H-bonds between the SCPN and the solvent, those with AMBER and OPLS exhibit the opposite trend (Figure 6C). In the case of the GROMOS-modified force fields, the impact of the mass repartition/increase does not seem to follow a clear tend (Figure 6D), with maximum and minimum values for HMR and H2H7, respectively. Regarding the interaction between CPs and lipid heads, the simulations with the different GROMOS parameterizations, as well as that with AMBER, provide the lowest numbers of H-bonds (Figure S5). HIE (trajectories H2D, H2Q, and H27H) also has a significant impact, leading to a higher number of H-bonds, while the results for the HMR and HMR_w_ trajectories are equivalent to those with standard GROMOS.

The lifetime of the H-bonds (Figure S6) between inner CP pairs is higher than for the CPs at the edge of the SCPN, for all trajectories. AMBER provides the largest lifetimes, while GROMOS—and in particular the simulations using the modified parameterizations with larger time steps—gives significantly shorter lifetimes. A similar trend takes place for the lifetime between CPs and water molecules, where the largest value corresponds to CHARMM, followed by OPLS and AMBER. The corresponding lifetimes for the GROMOS parameterizations are less than half of that using CHARMM. The lifetime for the H-bonds between CPs and lipids also exhibits a similar behavior. In this case, the maximum value corresponds to OPLS, followed by CHARMM and AMBER. The simulations using the GROMOS parameterizations provide the shortest lifetime values for this interaction. HMR and HIE modifications do not significantly affect the lifetime of any of the H-bond determinations.

### 3.2. Force Field Dependence of the Structure of the Nanopores Formed by the D,L-α-SCPN

**Figure 7:**
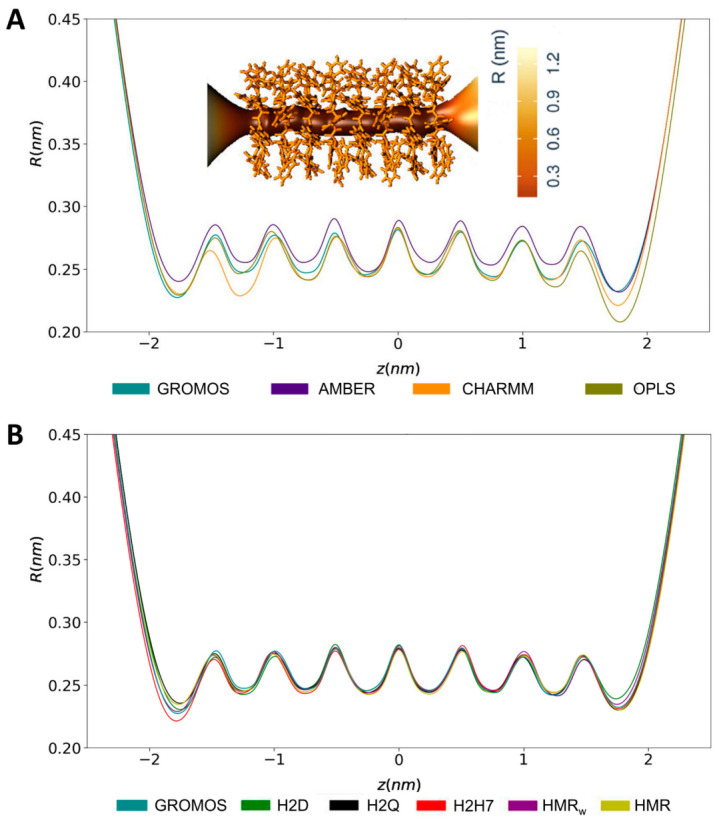
Inner radius (nm) of the SCPN calculated with CHAP [72] throughout the MD simulations, using the different the force fields (**A**) and modified parameterizations of GROMOS based on hydrogen mass repartition/increase (**B**).

The SCPN structure creates a stable open pore across the lipid membrane. The radius of the cavity along the nanopore axis exhibits a regular pattern, with minima at the level of all of the CP planes, and maxima in the inter-CP regions (Figure 7), in agreement with previous works [28]. The radius observed for the AMBER trajectory was slightly larger than for other force fields, including those with the modified GROMOS parameters, as anticipated by the longer distances from the Cα to the center of each CP (Figure 3).

**Figure 8:**
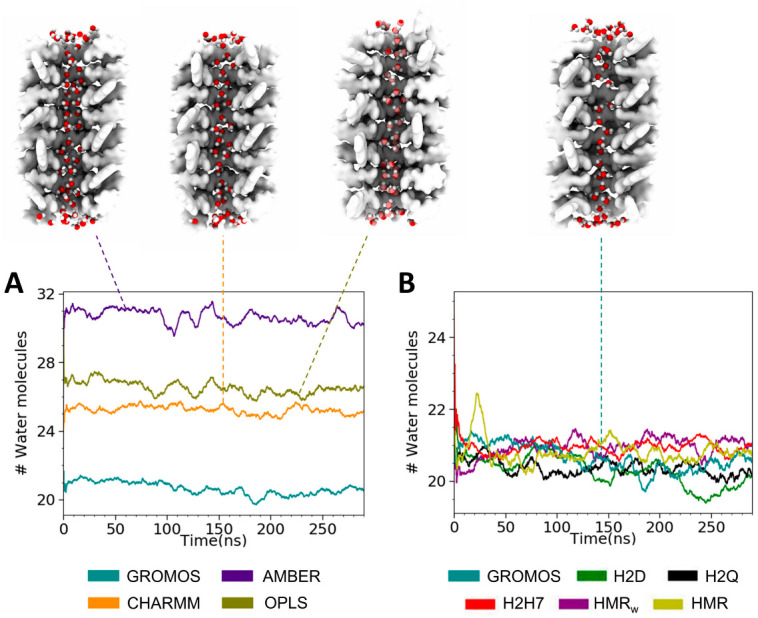
Evolution of the number of water molecules inside the SCPN along the trajectory, for each force field (**A**), and for the modified parameterizations of GROMOS based on hydrogen mass repartition/increase (**B**).

The penetration of water inside the channel presented considerable differences depending on the force field employed (Figure 8A). The water location inside this type of nanotube has been suggested to follow a (1–2)_n_ profile (with one molecule in the CP planes and two molecules between CPs) [79], giving a total of 24 water molecules within the cavity. The simulations using the GROMOS force field, including those with modified topologies, fit this pattern better than those with OPLS, CHARMM, or AMBER, which incorporate several extra water molecules inside the cavity. Notably, the simulations performed using the AMBER force field included an average of ~31 water molecules inside the SCPN along the whole trajectory. The higher occupation of water molecules inside the channel is probably related to the larger inner radius exhibited by the SCPN when it is simulated with the AMBER force field. In contrast to what was expected, and was observed for the simulations based on GROMOS, the water profile observed for the simulations with AMBER was (2–3)_n_ (Figure 8A). In any case, it should also be noted that the water model was not the same for all of the simulations, since it was selected based on what was employed in the original force field parameterizations.

**Figure 9:**
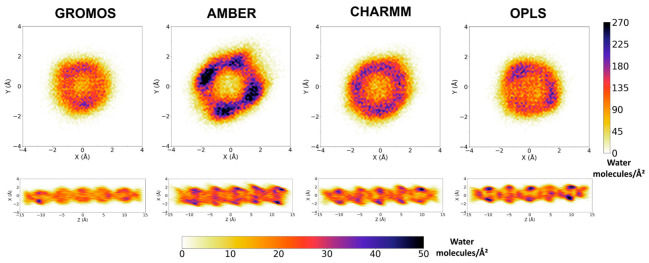
Two-dimensional positional probability distributions of water molecules determined along the last 220 ns of the trajectories, represented as an orange–blue color gradient for the force fields indicated in the labels. Only the region corresponding to the 6 inner CPs is considered for this analysis. Analogous profiles corresponding to the GROMOS-modified force fields are represented in Figures S7 and S8. Note that the longitudinal perspective (in the XZ plane) represents the projection in two Cartesian dimensions of the density of water molecules contained in a cylinder-shaped volume; thus, the middle area of the image (around X = 0) is affected by the low-density central region of the channel, while the edges of the projection only have the contribution of the cylinder contour.

The observed trend in the number of water molecules filling the SCPNs is also reflected in the 2D density maps (Figure 9, Figures S7 and S8) of the confined solvent. The water inside the channels reveals a different pattern for each force field, suggesting higher densities for AMBER and lower densities for GROMOS, in both the transversal and longitudinal directions. No significant differences in the density profiles can be appreciated between the modified force fields, in agreement with the similar numbers and locations of confined water molecules found inside the channels for the corresponding MD simulations.

The longitudinal perspectives in Figure 9 show that the distribution of water molecules throughout the channel was more homogeneous for the simulations using AMBER, CHARMM, and OPLS than for those corresponding to the GROMOS parameterizations. This result is consistent with the images shown in Figure 8. The density of water for the end CPs of the nanotubes was similar for all of the force fields, while the simulations using GROMOS contained fewer water molecules in the central region of the structure. This inhomogeneous distribution of the solvent molecules along the channel explains the important differences in the total numbers of water molecules contained in the nanopores (Figure 8). This feature could be used for experimental validation of force fields, when the density of water molecules throughout an SCPN can be measured.

### 3.3. Force Field Dependence of D,L-α-SCPN Diffusion Properties

One of the critical aspects of the hydrogen mass manipulation strategies employed in the present paper is related to the effect they can have on the internal dynamics of the molecule, as well as on its global displacement. Feenstra et al. [44] pointed out that increasing the total system mass by selectively changing atomic masses could be equivalent to scaling down the total simulation time. Thus, the reported simulation lengths are offset by a factor that is related to the ratio between the masses of the original and the manipulated systems. This was the reason why Feenstra et al. proposed the HMR method, prescribing the repartitioning of mass among the atoms in the system, so that its total mass does not change. However, when using UA force fields such as GROMOS, the total mass increase of the molecule upon HIE of just the polar hydrogens was relatively small, or even null in some cases where no polar hydrogens were present (POPC, for instance). For the CPs used in this work, the total mass gain for the H2D, H2Q, and H2H7 simulations represented 3.4%, 11.4%, and 14.6% of this solute molecule, respectively. Additionally, the mass increase in the polar hydrogens can be supported by experiments using different isotopic contrasts, which are typically assumed—and in some cases, have even been demonstrated—to provide identical thermodynamic and structural properties [80,81]. This perfectly justifies the use of deuterium instead of hydrogen for the polar hydrogen atoms. The use of quadium is an extrapolation of the hydrogen-to-deuterium exchange by an additional factor of two in the mass of these atoms. Finally, H7 was employed as a limiting and less realistic case to assess the impact of a significantly larger mass increase. Out of the theoretical and conceptual discussion, the evolution of the tilt angle of the whole SCPN and the lateral displacement profiles were determined, since these properties are expected to be especially sensitive to the mass manipulation. The tilt angle experienced a significant evolution, with a progressive growth that typically reached a plateau after 100 ns (Figure S9). Significant fluctuations were still present after this time, as can be appreciated mainly for the simulation with OPLS. The tilt of the simulations with the different GROMOS parameterizations is comparable to those observed for OPLS, CHARMM, and AMBER. The tilt angles of the individual CPs are highly correlated with one another (Figure S9), as shown by their synchronized time evolution, and also by the PCA analysis based on the correlation matrix of the tilt angle per CP unit. The result of this PCA analysis shows that more than 90% of the CP tilts can be represented just by a single component for the unmodified force fields, while the HMR and HMR_w_ simulations require at least two components to properly describe the tilting of all of the CPs. Overall, this analysis reveals slight dynamic differences in the relative movement of the CPs within the SCPN. The tilt angle of all the CPs is synchronized for all of the force fields, as well as for the different GROMOS parameterizations, and only the HMR approaches seem to be affected.

**Figure 10:**
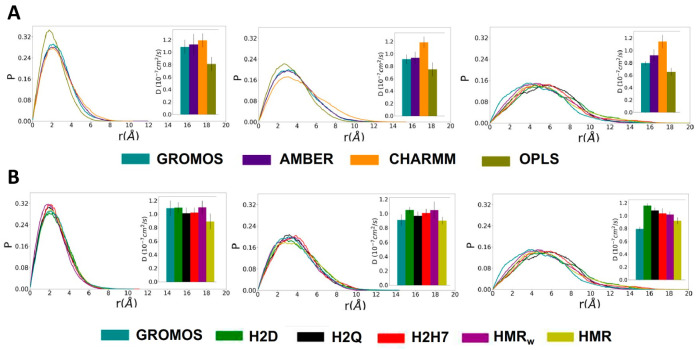
Lateral displacement probability (P) for the SCPN in different time windows for the classical (**A**) and modified (**B**) force fields. The corresponding distributions were fitted to the two-dimensional random walk equation to obtain the corresponding diffusion coefficients, represented in the insets of each graphic.

The lateral displacement distributions of the SCPN are very similar for GROMOS and AMBER, while the simulations with OPLS present a narrower distribution with lower displacements, and CHARMM shows the opposite behavior (wider distributions with larger displacements) for equivalent time windows (Figure 10A). The distributions for the simulations with the modified GROMOS parameters do not show significant differences between them (Figure 10). Thus, neither the HIE or the HMR methods seem to have a serious effect on the global diffusion of the SCPN. The fitting of these curves to the two-dimensional random walk equation leads to an estimation of the lateral diffusion coefficients (see Table S1 and Figure 10). These results confirm that the diffusion of the nanotubes with OPLS and CHARMM force fields is systematically lower and larger, respectively, than for AMBER and for the GROMOS parameterizations, for all of the time windows. The diffusion coefficients for HMR_w_ are slightly lower than for the other GROMOS approaches. Overall, even when the diffusion coefficient proved to be sensitive to the force field, the obtained values were very similar for all of the HMR and HIE parameterizations.

## 4. Conclusions

A critical comparison of different classical force fields, including AMBER, CHARMM, OPLS, and GROMOS, was carried out for MD simulations of an SCPN formed by *c*-[(*L*-Trp-*D*-Trp-)_4_] and inserted into a POPC bilayer. The performance of these force fields has been extensively tested on linear peptide and protein systems, but it remained to be tested on this kind of highly constrained CP. Significant differences (larger differences in the values than in the uncertainties) were observed from the analysis of our simulations. The diameter of the cylindrical cavity was observed to be larger for the simulation using AMBER and shorter for the simulation using CHARMM, while OPLS and GROMOS exhibited a similar cavity size. The average number of water molecules found within the nanopores was also very different for the different force fields. The nanopores simulated with AMBER hosted approximately 31 solvent molecules, while those with OPLS, CHARMM, and GROMOS contained ~27, ~25, and ~21 water molecules, respectively. This variation is very important, since it is expected to seriously affect the transport properties of the channel. In addition to the cavity diameter and the number of hosted water molecules, the lateral diffusion of the nanotube was also different for the different force fields. The fastest lateral diffusion coefficient was found for CHARMM, while the slowest was observed for the simulation with OPLS. GROMOS and AMBER exhibited similar lateral diffusion coefficients. The results were much more similar for other properties, such as the tilting angle, coordinate movements of the CPs, and different H-bond contributions. A significant difference was also observed in the angle between the Cα and the plane of the CP ring. The simulations with GROMOS showed the lowest angles, while the largest were observed for the simulations with OPLS. The differences in these angles seem very small, but they could contribute to changes in different structural or dynamic properties. The observed discrepancies were qualitatively similar to those observed in previous works aiming to compare the MD trajectories of biomolecular systems using different force fields [82,83,84]. It is difficult to find a single source for those differences, which were probably due to combination of differences in partial charges, van der Waals parameters, water models, and even the implicit or explicit representation of non-polar hydrogen atoms; however, it is very important to consider them for future experimental validation of the force fields, and also because they are expected to seriously affect the use of these systems for practical applications.

Furthermore, several modifications were performed on GROMOS54a7 in order to increase the time step used for the integration of the motion equations and, thus, to speed up the simulations. The HMR and HIE methods were tested. The second method was employed using deuterium (H2D), quadium (H2Q), and hydrogen 7 (H2H7) as isotopic elements. Quadium proved to be the best isotope to replace the polar hydrogens, since the simulations with deuterium were not stable, with a time step of 6 fs, and H7 does not allow extension of the time step further than this amount. These modifications allowed a significant time-step increase up to 5–6 fs, without serious changes in the structural and dynamical properties of the simulated peptide nanotubes when compared with the unmodified force field. Accelerating the simulations by a factor of three without losing resolution is definitely interesting considering the size of these systems and the time scale of molecular mechanisms required for some applications proposed for them.

Not enough experimental information is currently available to conclusively decide which of the employed force fields or GROMOS parameterizations is better than the others for computational studies of SCPNs. On the other hand, the methods employed to speed up the simulations are especially well suited to the GROMOS force field due to its special treatment of the non-polar hydrogen atoms. The quantitative descriptors reported in this work are intended to guide the design of future experiments aimed at validating the application of classical force fields to simulate SCPNs. Some properties that have yielded statistically significant differences between force fields, and are important for future experimental validation, are summarized in Table S2. Once the required experimental information is available, all of the force fields, including GROMOS, should evolve to converge with experiments. Thus, the application of the HMR and HIE methods is not limited by any eventual update in the force fields.

## Figures and Tables

**Table 1:** Summary of the time steps employed in each MD simulation, as well as the corresponding masses of the polar atoms in the system. Note that there are no polar hydrogens in GROMOS POPC lipids, so the only affected atoms belong to the CP.

	AMBER	CHARMM	OPLS	GROMOS	H2D	H2Q	H2H7	HMR	HMR_w_
**Time step/fs**	2	2	2	2	5	6	6	6	7
**Water model**	TIP3P	TIP3P	TIP4P	SPC	SPC	SPC	SPC	SPC	SPC
**Polar H/Da**	1.008	1.008	1.008	1.008	2.014	4.026	7.053	4.032	4.032
**Polar C/Da**	12.010	12.011	12.011	12.011	12.011	12.011	12.011	8.987	8.987
**Water O/Da**	16.000	16.000	16.000	15.999	15.999	15.999	15.999	15.999	9.951
**Water H/Da**	1.008	1.008	1.008	1.008	1.008	1.008	1.008	1.008	4.032

## Data Availability

All initial coordinates, topologies, and mdp files needed for reproducing these simulations are available in Zenodo (https://zenodo.org/record/6334053#.YiXPsxso9H4, accessed on 10 February 2022). Further inquiries can be directed to the corresponding author.

## Supplementary Materials

The following supporting information can be downloaded at https://www.mdpi.com/article/10.3390/ijms23063158/s1.
