# Supplementary material for: Molecular Dynamics Simulations of Transmembrane Cyclic Peptide Nanotubes Using Classical Force Fields, Hydrogen Mass Repartitioning, and Hydrogen Isotope Exchange Methods: A Critical Comparison
Source: Int J Mol Sci. 2022 Mar 15;23(6):3158. doi: 10.3390/ijms23063158 (PMC8951607; doi:10.3390/ijms23063158)
Supplement: Supplementary file 1 [file ijms-23-03158-s001.zip › ijms-1624593-supplementary.pdf]

Molecular dynamics simulations of transmembrane cyclic peptide nanotubes using classical force fields, Hydrogen Mass Repartitioning and Hydrogen Isotope Exchange methods: a critical comparison

Daniel Conde[1,2], Pablo F. Garrido[2], Martín Calvelo[1,3], Ángel Piñeiro[2,], Rebeca Garcia-Fandino[1,*]*

[1]Departamento de Química Orgánica, Center for Research in Biological Chemistry and Molecular Materials, Universidade de Santiago de Compostela, Campus Vida s/n, E-15782 Santiago de Compostela, Spain [2]Departamento de Física Aplicada, Facultade de Física, Universidade de Santiago de Compostela, E-15782 Santiago de Compostela, Spain). [3]Departament de Química Inorgánica i Orgànica and Institut de Química Teòrica i Computacional (IQTCUB), Universitat de Barcelona, Barcelona 08028, Spain

Supporting Information

A.

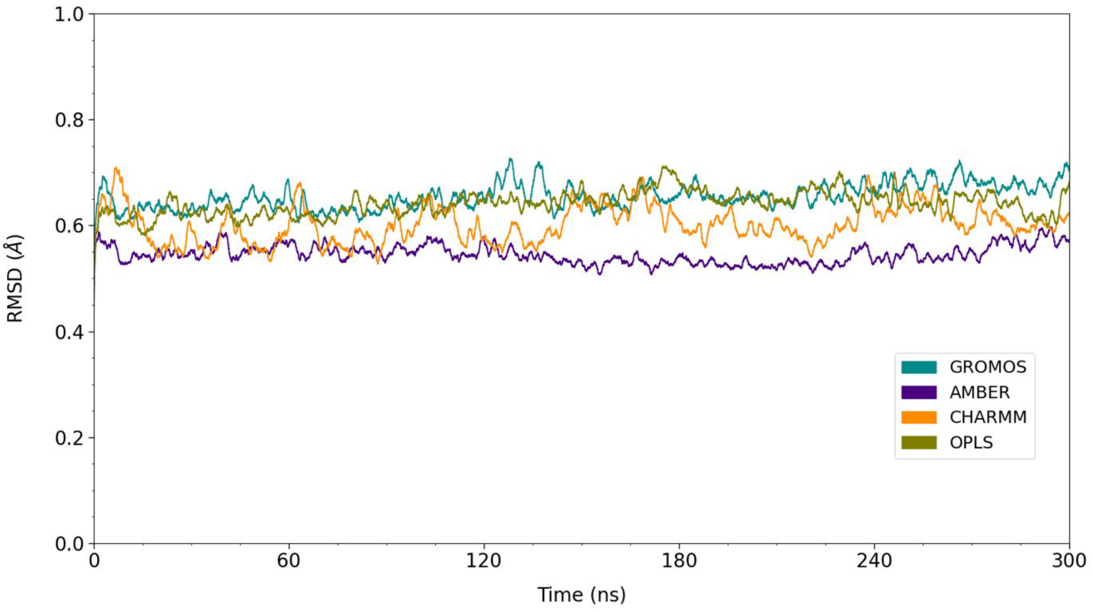

B.

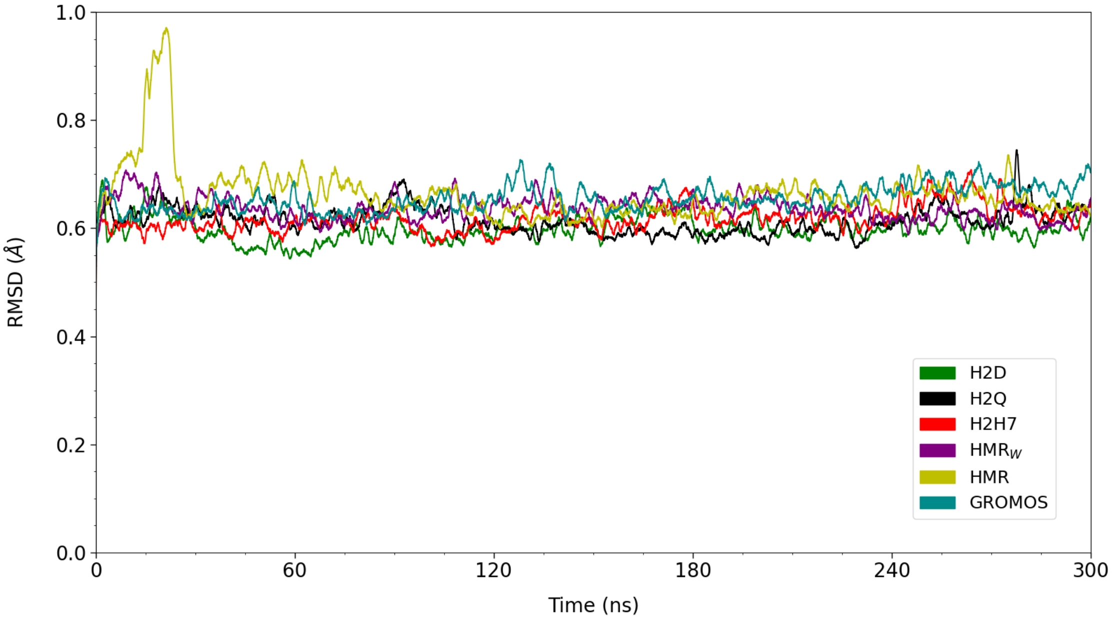

Figure S1.- RMSD of the SCPN backbone along the whole trajectory, for the classical (**A**) and the modified force-fields (**B**), with respect to the initial structure (after minimization).

A.

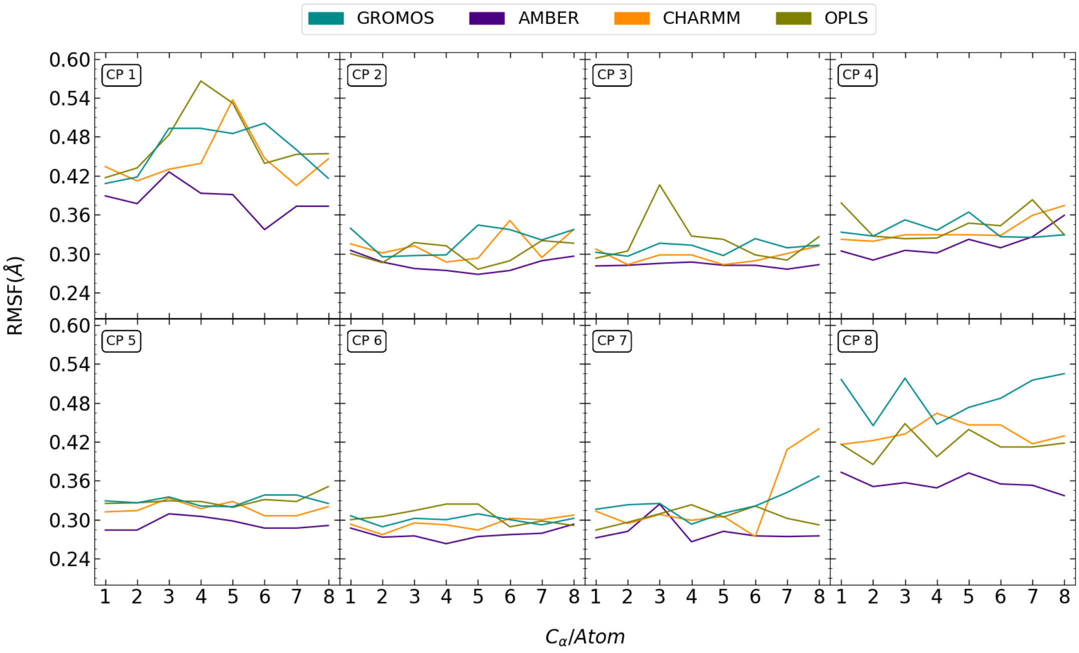

B.

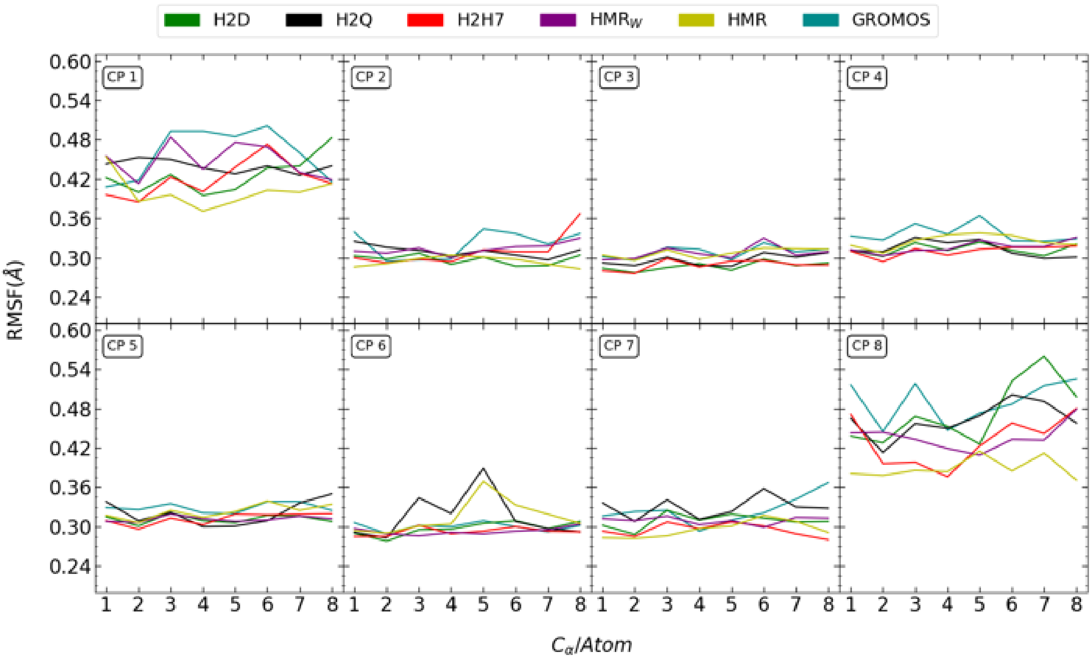

Figure S2.- RMSF of each CP backbone along all the trajectory, for the classical (**A**) and the modified force-fields (**B**), with respect to the initial structure (after minimization).

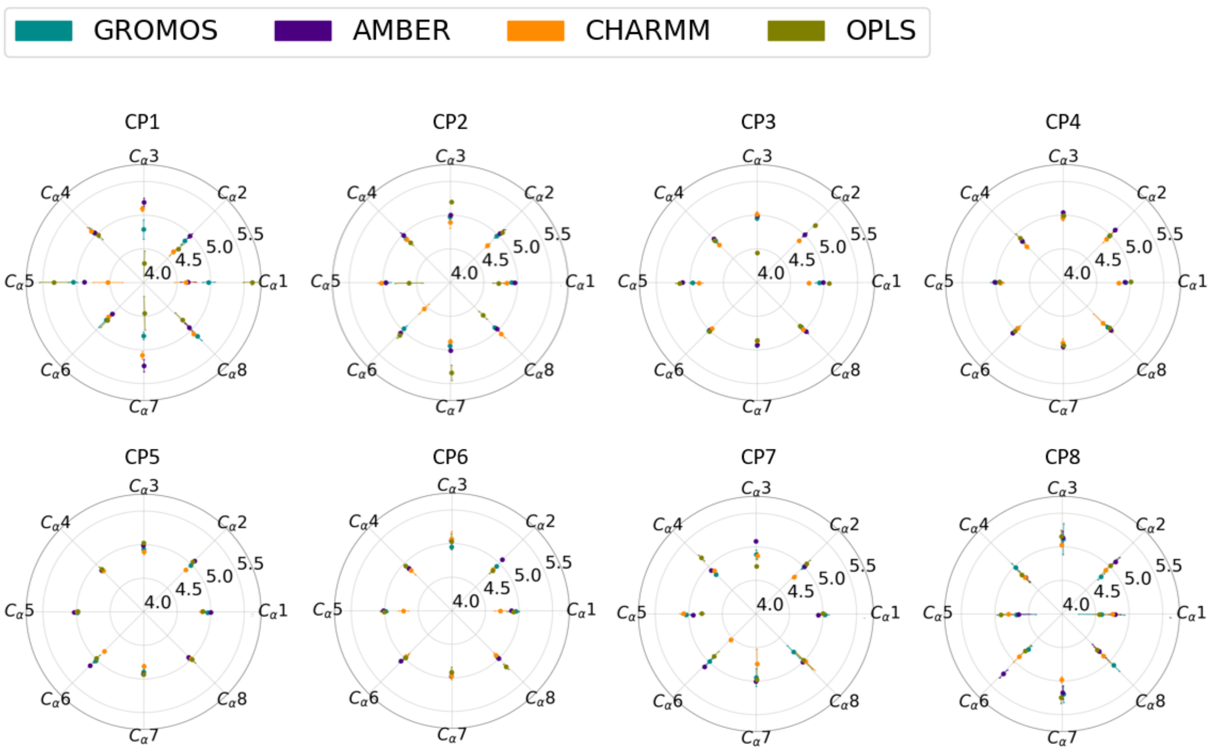

B.

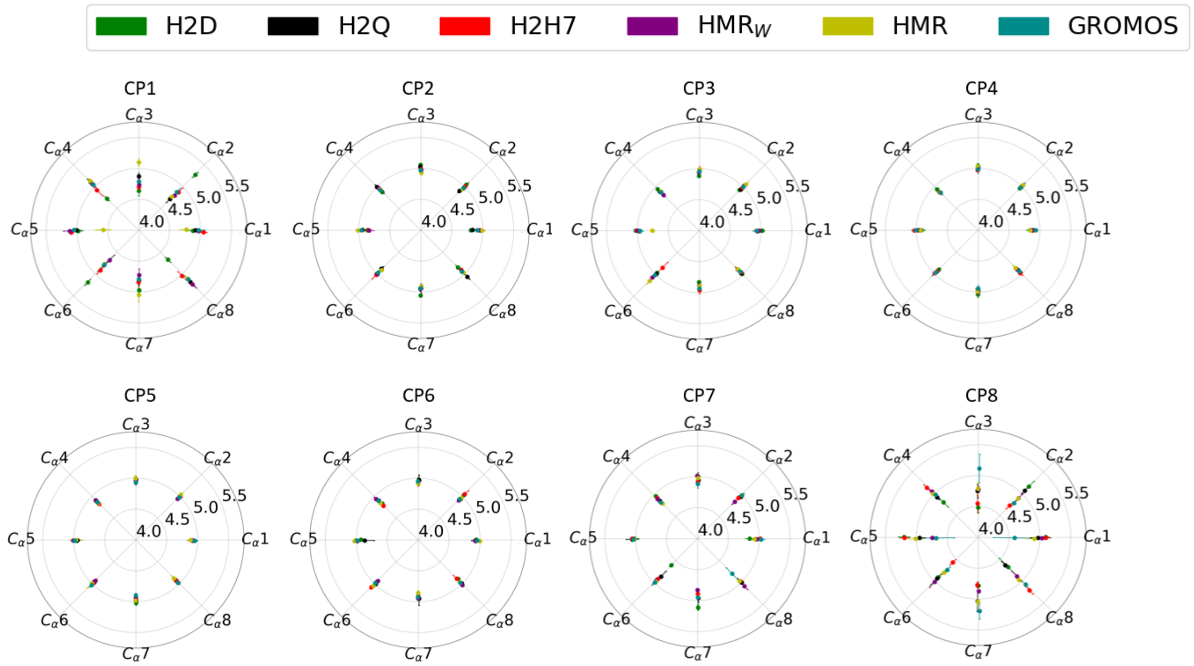

Figure S3.- Projection of each Cα on the plane defined by each CP ring, calculated as an average over the whole trajectory and represented at its correspondent distance to the

geometric center. The radial lines correspond to the ideal location of each Cα. Deviations from these lines represent the average angular displacement of the Cα. Each color represents a different force-field, as indicated in the legends. Results for the unmodified OPLS, AMBER, CHARMM and GROMOS force fields are represented in **A** while those for HMR and HIE parameterizations of GROMOS are plotted in **B**. The classical GROMOS force-field is included in both representations since it is used as a reference for the modified force-fields.

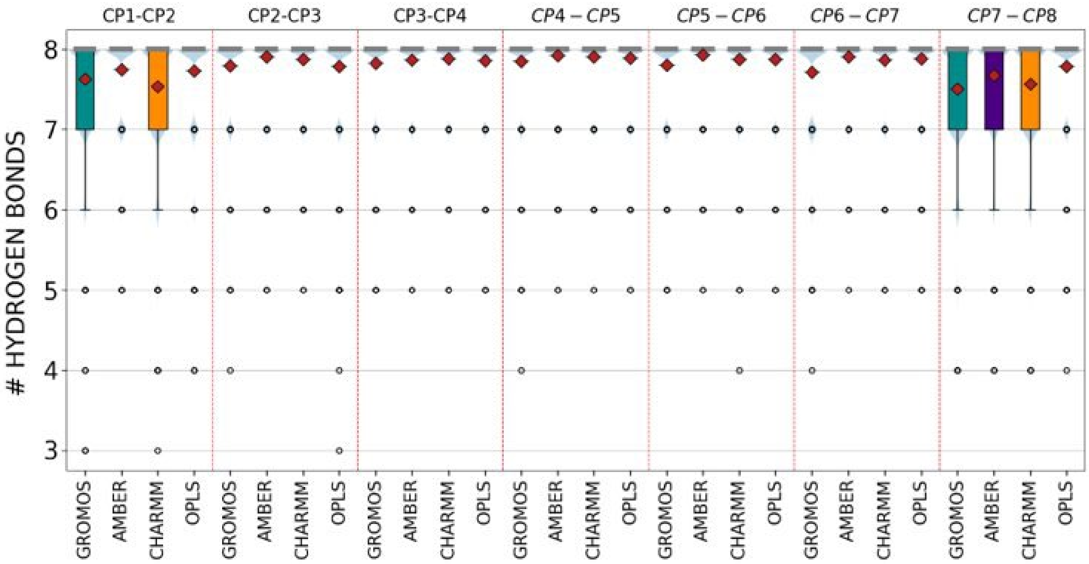

B.

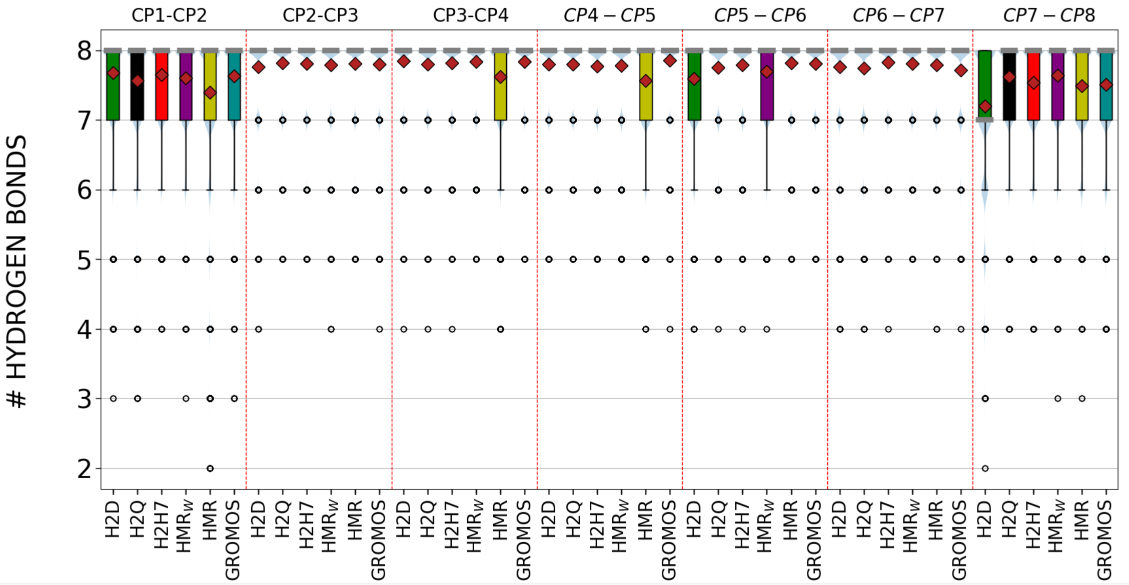

Figure S4.- H-bonds between the different CPs, averaged over the whole trajectory for the classical force-fields (**A**) and the GROMOS-modified force-fields (**B**). The grey line represents the median and the red square the mean of the distributions. The size of the squares includes data within the second and third quartiles, so that 50% of the data are within the corresponding box. The whiskers include data of the first and fourth quartiles.

A.

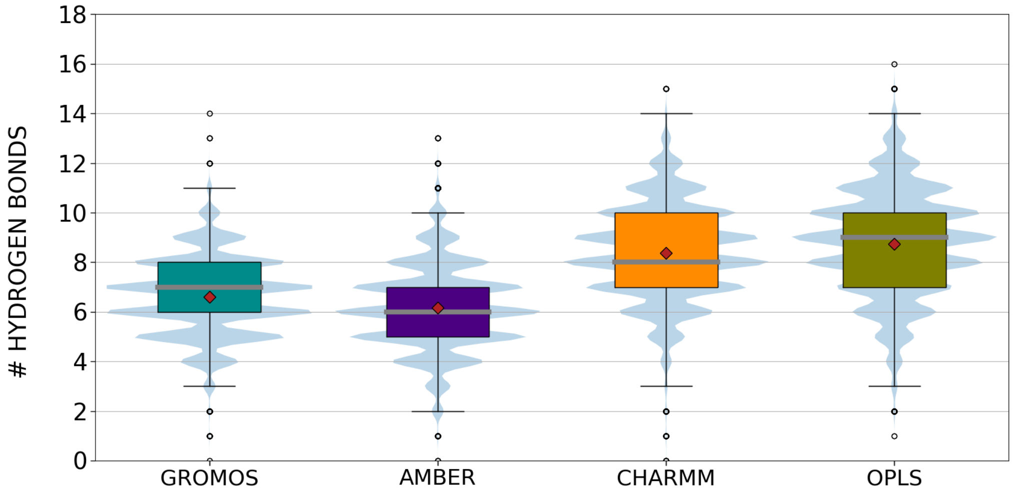

B.

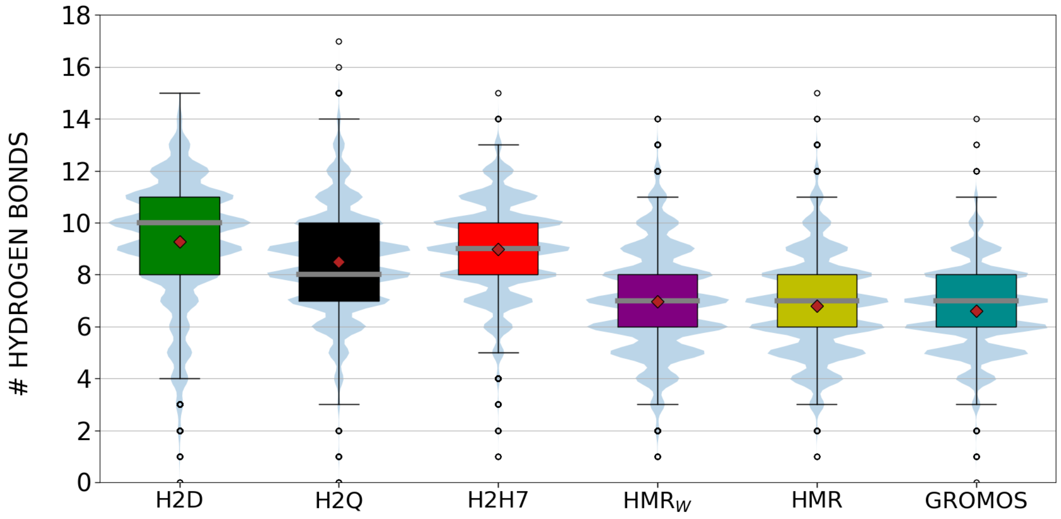

Figure S5.- H-bonds between the CPs and the lipids, averaged over the whole trajectory for the classical force-fields (**A**) and the GROMOS-modified force-fields (**B**). The grey line represents the median and the red diamond the mean of the distributions. The size of the boxes includes data within the second and third quartiles, so that 50% of the data are within the corresponding box. The whiskers include data of the first and fourth quartiles.

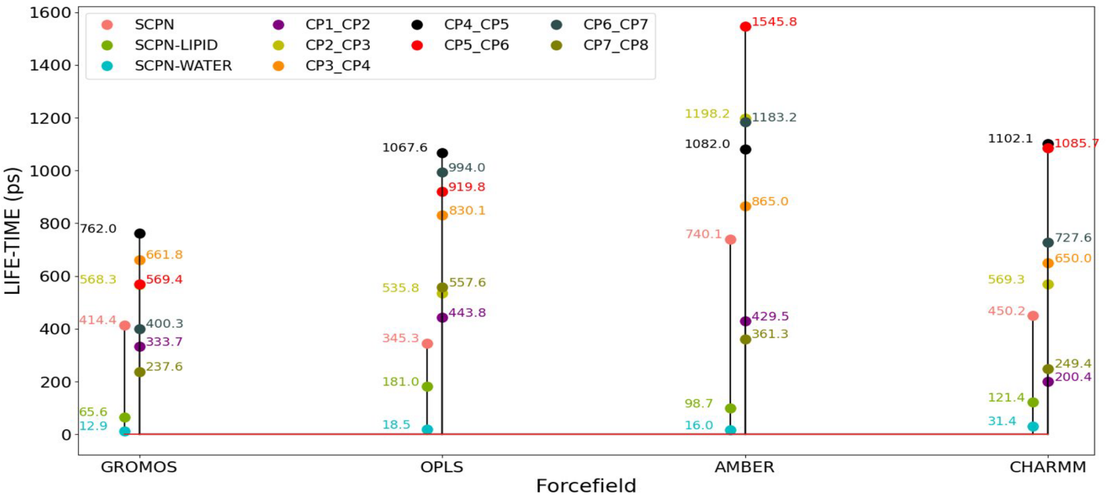

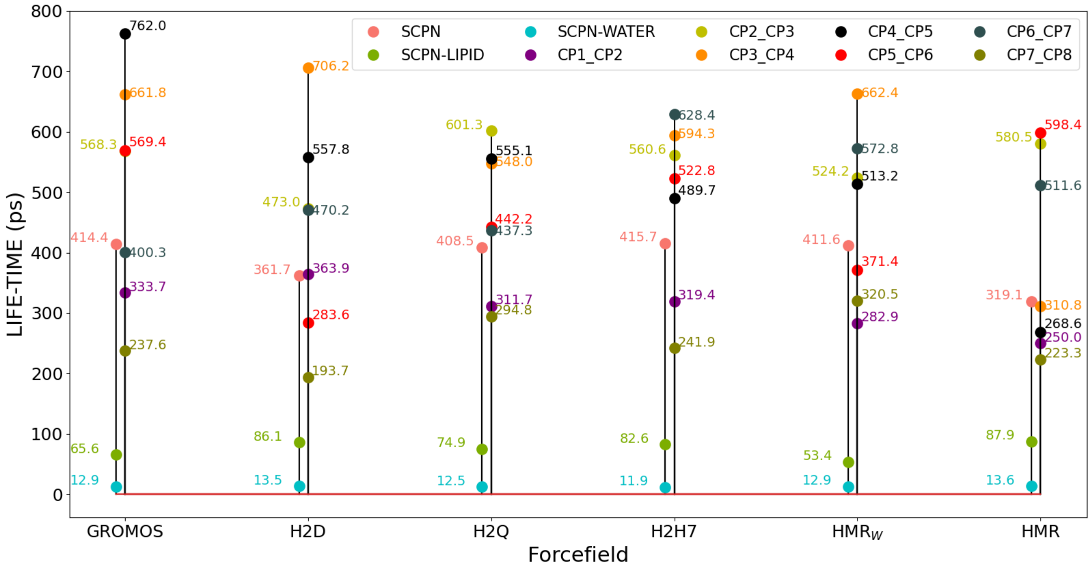

Figure S6.- Lifetimes obtained for different H-bond contributions, as indicated in the legend.

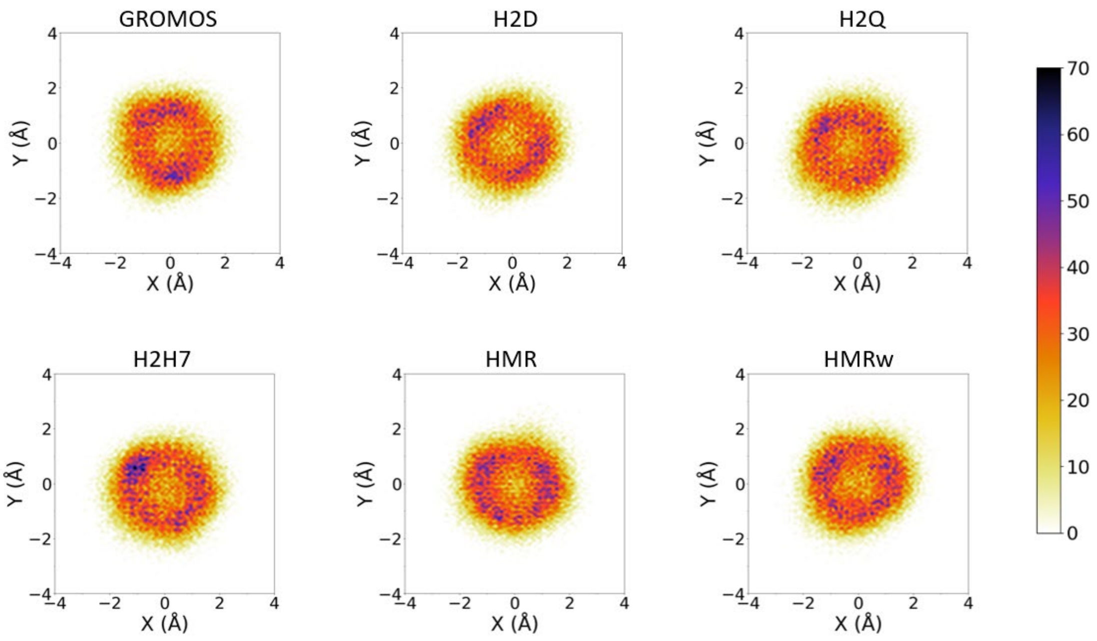

Figure S7.- Two-dimensional positional probability distributions on the XY plane for the modified force fields (and GROMOS, as a reference). Just the region between the 6 inner CPs is considered.

A.

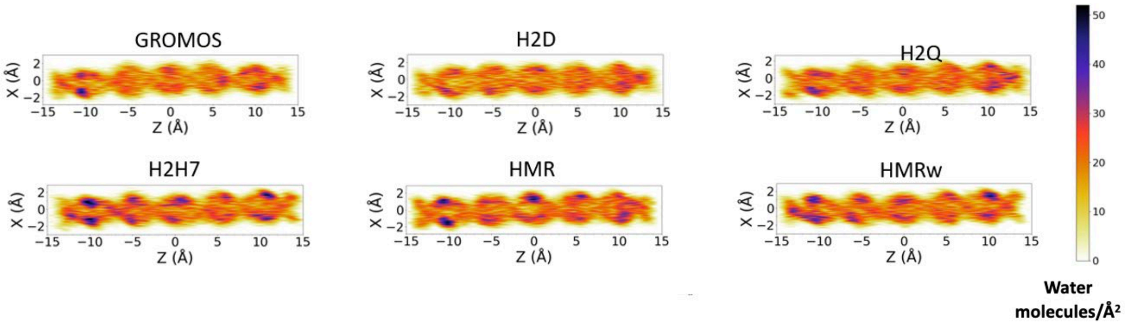

B.

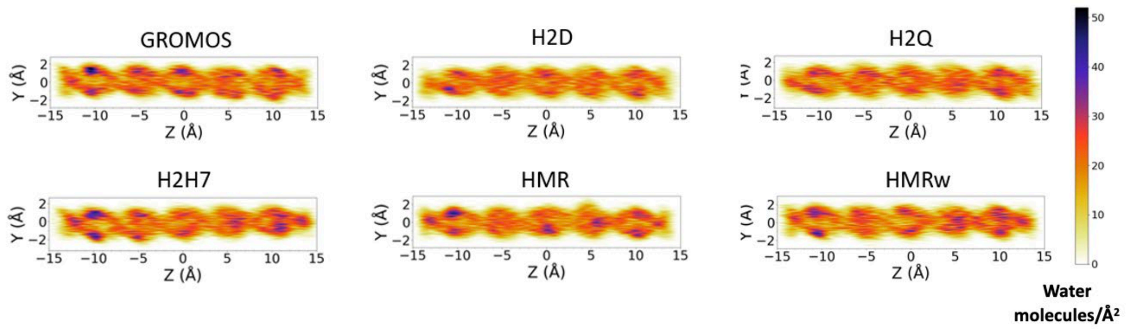

Figure S8.- Two-dimensional positional probability distributions on the XZ and YZ planes for the modified force fields (and GROMOS, as a reference). Just the region between the 6 inner CPs is considered.

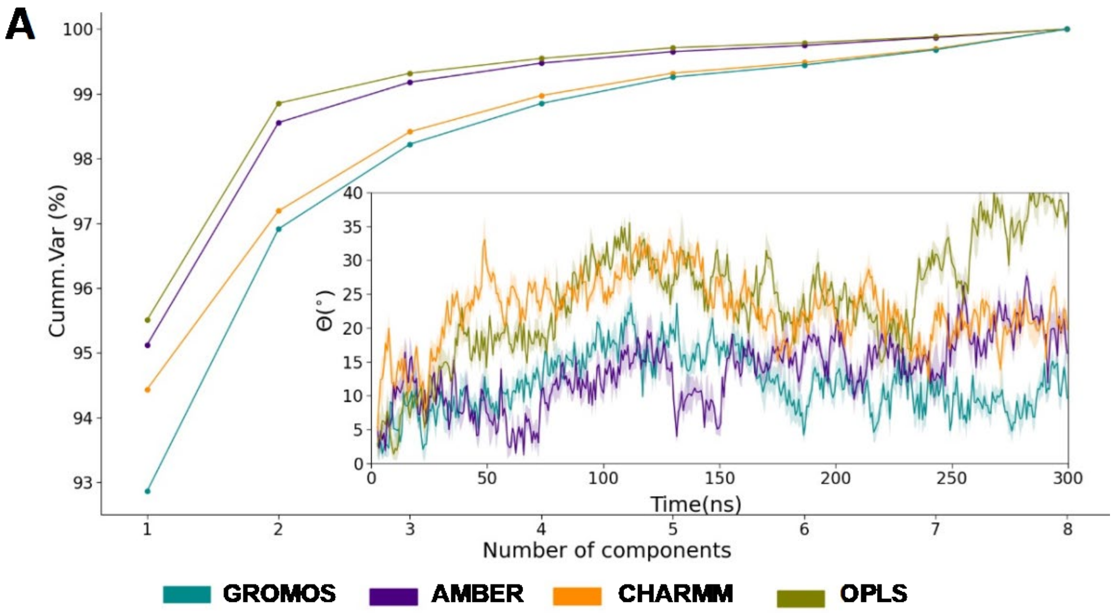

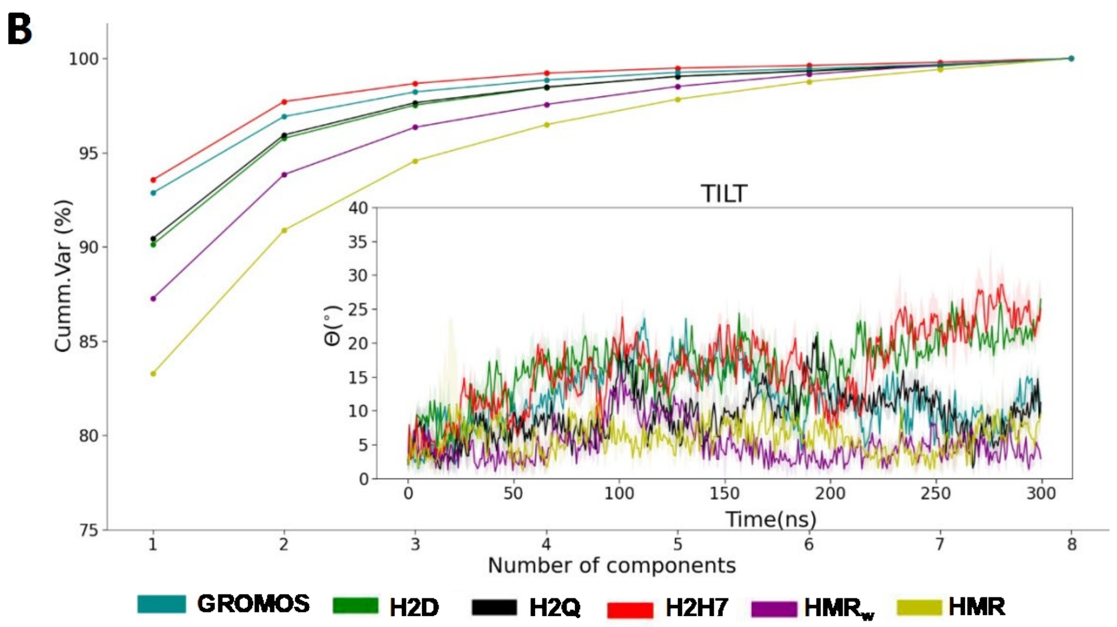

Figure S9.- Eigenvalues obtained from the principal component analysis for the tilt angle (see methods section) per CP subunit in each simulation. The average tilt angle for each SCPN as a function of time is also included.

Table S1.- Diffusion coefficients (x 10^{-7} cm^2/s) calculated from the fitting of the two-dimensional random walk equation to the displacement probabilities (see Fig. 10).

	**2 ns**	**5 ns**	**10 ns**
**AMBER**	1.12±0.17	0.934±0.098	0.919±0.096
**CHARMM**	1.19±0.11	1.185±0.090	1.142±0.011
**OPLS**	0.811±0.11	0.748±0.11	0.654±0.065
**GROMOS**	1.086±0.086	0.910±0.077	0.793±0.037
**H2D**	1.095±0.049	1.048±0.030	1.158±0.021
**H2Q**	1.064±0.055	0.998±0.042	1.080±0.046
**H2H7**	1.019±0.074	1.007±0.058	0.906±0.077
**HMR**	1.099±0.098	1.05±0.12	1.019±0.043
**HMR_w**	0.89±0.12	0.899±0.053	0.921±0.053

Table S2.- Summary of the properties with statistically significant differences between forcefields, important for future experimental validation. H / S indicates the force field that had significantly higher/smaller values than the rest.

	Rigidity	Tilt Angle	H-bonds SCPN-Lipids	H-bonds SCPN-Water	Distance between CPs	#Water molecules inside the SCPN	Inner radius	Lateral displacement
**AMBER**	H	-	S	-	H	H	H	-
**GROMOS**	-	S	S	-	-	S	-	-
**CHARMM**	-	-	H	S	-	-	-	H
**OPLS**	-	H	H	-	-	-	-	S
